# Exploring capabilities of elemental mass spectrometry for determination of metal and biomolecules in extracellular vesicles

**DOI:** 10.1007/s00216-023-05056-0

**Published:** 2023-11-24

**Authors:** Jaime Martínez-García, Alicia Villa-Vázquez, Beatriz Fernández, Héctor González-Iglesias, Rosario Pereiro

**Affiliations:** 1 Department of Physical and Analytical Chemistry, University of Oviedo, Julian Clavería 8, 33006 Oviedo, Spain; 2 Dairy Research Institute of Asturias, Spanish National Research Council (IPLA-CSIC), Villaviciosa, Spain

**Keywords:** Extracellular vesicles, Exosomes, Isolation methods, Single-vesicle analysis, Mass spectrometry, ICP-MS

## Abstract

Extracellular vesicles (EVs) are increasingly recognized as crucial components influencing various pathophysiological processes, such as cellular homeostasis, cancer progression, and neurological disease. However, the lack of standardized methods for EV isolation and classification, coupled with ambiguity in biochemical markers associated with EV subtypes, remains a major challenge. This *Trends* article highlights the most common approaches for EV isolation and characterization, along with recent applications of elemental mass spectrometry (MS) to analyse metals and biomolecules in EVs obtained from biofluids or in vitro cellular models. Considering the promising capabilities of elemental MS, the article also looks ahead to the potential analysis of EVs at the single-vesicle and single-cell levels using ICP-MS. These approaches may offer valuable insights into individual characteristics of EVs and their functions, contributing to a deeper understanding of their role in various biological processes.

**Graphical Abstract:**

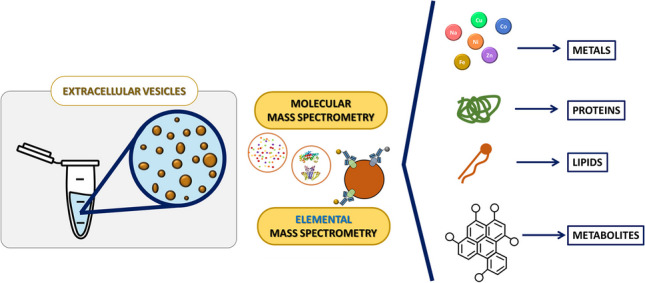

## Introduction

The study of extracellular vesicles (EVs) is gaining importance as they are increasingly recognized as promising samples in biomedical research. EVs represent a novel paradigm of cell-to-cell communication, and the study of target analytes in the EVs population can provide advanced knowledge to understand the bidirectional communication between cells and their complex microenvironment [1]. EVs are particles with a lipid bilayer membrane that contain a wide variety of cargos (e.g., lipids, proteins, metabolites, sugars, and RNA) from the host cell. Despite the existence of various EVs subpopulations described in the literature, there is a lack of homogeneity in their classification. Ambiguity in biochemical markers associated with EVs subtypes and the absence of standardized methods to isolate EVs have remained the major bottleneck in addressing the heterogeneity and significance of EVs [2]. Based on their biogenesis and biophysical characteristics, EVs can be categorized into three groups: (i) exosomes, which are typically 40–160 nm in diameter and derived from intracellular endosomal compartments; (ii) microvesicles (100–1000 nm) produced by outward budding and pinching-off the plasma membrane; and (iii) apoptotic bodies (50–5000 nm), released as blebs by cells undergoing apoptosis [3].

EVs have been isolated and detected in various body fluids, such as blood, urine, saliva, and breast milk [4, 5]. Due to their dynamic production, EVs are now considered as valuable biomarkers for diagnosing and monitoring of pathologies such as cancer, neurodegeneration, and cardiovascular diseases [6, 7]. Furthermore, the study of EVs isolated from culture media of in vitro cellular models (cell culture supernatant, CCS) is proving to be interesting for treatment evaluation. Additionally, EVs are being proposed as therapeutic agents for regulating inflammation, promoting tissue regeneration, and serving as novel modulators for vaccination [8].

Research efforts are still necessary in several directions for biomedical application of EVs. First, it is necessary to ensure the isolation of pure EVs subpopulations by minimizing impurities from reagents or other sub-micron particles. Second, standardized methods for EVs isolation need to be developed, considering the challenge posed by the specific composition and physical properties of each fluid. EVs show a high heterogeneity even within a single subpopulation [9], with distinct abilities to induce complex biological responses. This emphasizes the necessity for appropriate biomarkers to identify specific EVs and efficient purification strategies. To unravel their heterogeneity, significant research efforts have been devoted to developing cutting-edge methods for the isolation and characterization of EVs [10].

This *Trends* article covers recent applications of elemental and molecular mass spectrometry (MS) for determining metals and biomolecules in EVs derived from biofluids or secreted by in vitro cellular models. The heterogeneity of EVs populations has hindered our understanding of their biogenesis, composition, bio-distribution, and functions; hence, the article focuses on both traditional and state-of-the art approaches developed for the isolation and characterization of EVs, taking into account their heterogeneous nature. The emphasis of this article is on elemental MS; therefore, the opportunities and challenges associated with employing inductively coupled plasma (ICP) MS are highlighted. However, molecular MS techniques have been more extensively applied so far and, therefore, a brief overview on the topic is also provided. In the “Outlook” section, a perspective is presented on analysis at the single-vesicle and single-cell EVs levels using elemental MS.

## Isolation and characterization of EVs from biofluids and cultured cells

**Fig. 1:**
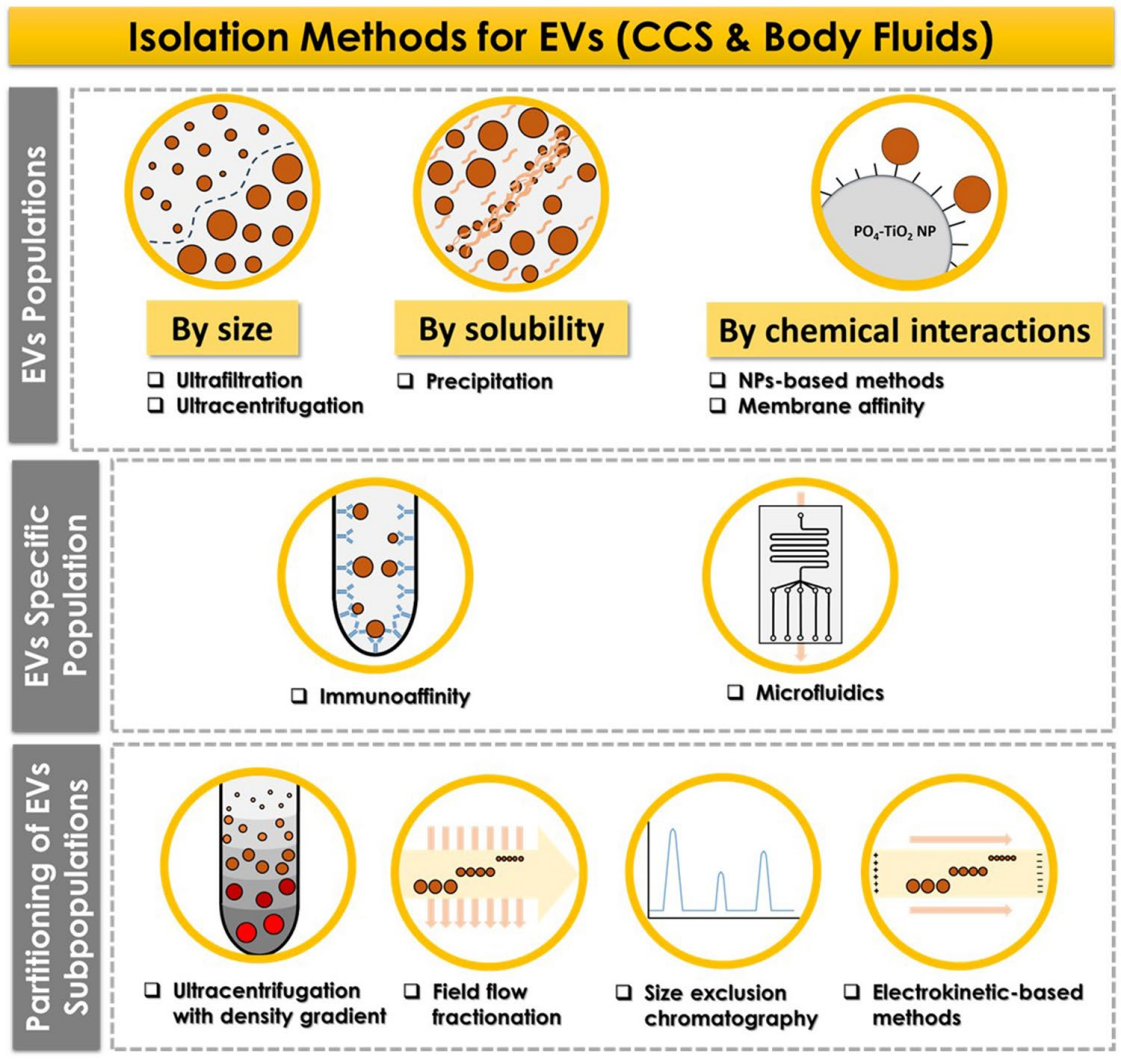
Methods commonly employed to isolate EVs from biofluids and CCS, categorized according to the level of isolation achieved: EVs populations, specific EVs populations, and partitioning of EVs subpopulations

Isolation and characterization of EVs is challenging and no standardized methods are currently available. The complexity, specific composition, and physical properties of each sample, along with the high heterogeneity of EVs, can impede obtaining reproducible results. This *section* provides a brief overview of both conventional and state-of-the art methods for EVs isolation and characterization. Figure 1 depicts a diagram illustrating methods for obtaining EVs, categorising them on the level of isolation achieved: (i) EVs populations, (ii) specific EVs populations, and (iii) partitioning of EVs subpopulations.

Traditional methods for the isolation of EVs, such as ultrafiltration, ultracentrifugation, and precipitation, allow for the isolation of EVs populations by applying specific cut-off criteria and are commonly employed for isolating EVs from biofluids and CCS, but they do not provide any additional information. Recently, nanoparticles (NPs)-based methods have been proposed to isolate EVs populations by leveraging the specific interaction between TiO_2_ NPs and the phosphate groups on the lipid bilayer of exosomes [11]. To obtain information on a specific EVs population, immunoaffinity or microfluidic devices can be employed. These approaches allow to tune the system depending on a specific label of the target EVs population [12]. Furthermore, advances in single-vesicle and single-cell EVs technologies, many of which are based on microfluidic devices and digital assays, enable the collection of information from a specific subpopulation [3, 13]. It is also possible to isolate EVs subpopulations by the partitioning of the sample in several fractions using ultracentrifugation with density gradient, field-flow fractionation (FFF), size-exclusion chromatography (SEC), or electrokinetic-based methods. These procedures can be tuned according to the desired subpopulation by adjusting parameters such as pore size, column volume or length in SEC, flow conditions in FFF, or electric fields in electrokinetic methods. Review articles are available providing detailed information about the main advantages and limitations depending on the desired application [4, 5, 14].

Once EVs isolation has been achieved, it is also challenging to verify the identity of EVs and to perform their characterization. Complementary techniques are employed to evaluate the efficacy of isolation methods and gain insights into the nature of EVs. Table 1 summarizes the most common techniques along with their inherent advantages and drawbacks. MS-based techniques are listed towards the end of Table 1 and deserve special attention due to their high sensitivity and capability to identify and determine the elemental and molecular composition of EVs. Molecular MS includes instruments with soft ionization sources with single or tandem MS/MS analysers [15, 16] and ICP-MS is an elemental technique able to provide both information on endogenous metal content and, after the proper labelling with elemental tags, also molecular information [17–19]. Special attention should be paid to mass cytometry, which combines flow cytometry with ICP-MS, allowing the simultaneous analysis of multiple parameters.

**Table 1:** Overview of the most frequently employed techniques for characterizing EVs, highlighting their key advantages in terms of the information they offer, as well as their limitations such as analysis requirements and challenging aspects

Technique	Advantages	Limitations
Electron microscopy	• High-resolution images for visualizing EV morphology, size, and internal structure • Examination of individual EVs	• Sample preparation, including fixation and staining, which can introduce artefacts • Time-consuming • Limited in throughput and quantification
Dynamic light scattering (DLS)	• Size distribution of EVs in a liquid suspension • Information on the hydrodynamic radius	• Limited resolution for smaller EVs • Cannot provide information on EVs morphology or internal structure
Nanoparticle tracking analysis (NTA)	• Size distribution and concentration of EVs in a liquid sample • Real-time tracking of individual particles	• Limited accuracy for smaller particles • Sensitive to sample properties (e.g., viscosity) • Limited information on EVs morphology or internal structure • Difficult to analyse heterogeneous samples
Atomic force microscopy (AFM)	• High-resolution images of EV morphology, size, and surface topography • Can operate in liquid environments	• Time-consuming and technically challenging • Limited throughput and quantification • Limited information on EV composition • Not suitable for high-throughput analysis
Raman spectroscopy	• Chemical information on EVs composition, including lipids, proteins, and nucleic acids • Non-destructive and label-free analysis	• Requires intense laser illumination, which may cause sample damage • Low signal-to-noise ratio for weak Raman scatterers • Data processing and interpretation • Not suitable for high-throughput analysis • Limited information on EVs size and morphology
Electrochemical sensing	• Quantitative analysis of EVs based on their electrochemical properties • Information on EVs concentration and surface markers	• Requires specific labels for EV detection • Limited sensitivity for low-abundance EVs • Limited information on EVs size, morphology, or internal structure • Not suitable for high-resolution imaging
Western blot	• Detects specific proteins present in EVs • Allows for comparison between samples	• Requires specific antibodies for target protein detection • Limited information on EV size or morphology • Semi-quantitative • Careful sample preparation
Enzyme-linked immunosorbent assay (ELISA)	• Allows quantitative measurement of specific EV-associated proteins or molecules • High sensitivity and specificity	• Requires specific antibodies or labels for target detection • Limited information on EV size
Immunofluorescence	• Detection and visualization of specific proteins or molecules on the surface or within EVs • Spatial information about the distribution and localization of specific markers	• Requires specific antibodies for the target molecules • Non-specific staining and high background signal • Quantitative analysis and multiplexing capabilities
Flow cytometry	• High-throughput analysis • Characterization of EVs populations based on their size, surface markers, and fluorescence properties • Quantitative data on the abundance and heterogeneity of EVs	• Size resolution (EVs below a certain size threshold may not be detected) • Requires proper calibration and controls to accurately determine EVs concentration • Multiplexing capabilities limited due to spectral overlap of fluorophores • Only surface antigens are typically studied
Molecular MS	• Analysis of EVs’ molecular content, including proteins and lipids • High-throughput (number of analytes per sample) • Identification and quantification of EVs components • Can uncover potential biomarkers • Information on post-translational modifications and protein isoforms	• Complex sample preparation: including EVs isolation and tryptic protein digestion • Extensive data analysis • Limited sensitivity; requires a relatively large amount of EVs for analysis (problems for the analysis of low-abundance proteins or lipids) • Obtaining quantitative information can be challenging due to variations in sample preparation
Mass cytometry	• Simultaneous analysis of multiple parameters on EVs at the single-particle level, including surface markers and intracellular proteins	• Requires extensive panel design and optimization • Technically challenging • Sample preparation may affect the detection of certain epitopes
Inductively coupled plasma MS (ICP-MS)	• Quantification of elements present in EVs • MNPs can be used for labelling or tracking purposes • High sensitivity • Accurate quantitative analysis	• Without labelling: specific to endogenous elemental analysis (does not provide information about other EVs components) • With labelling: selection of the most adequate label, which may alter their properties or introduce non-specific interactions

## Determination of target analytes in EVs by mass spectrometry

**Fig. 2:**
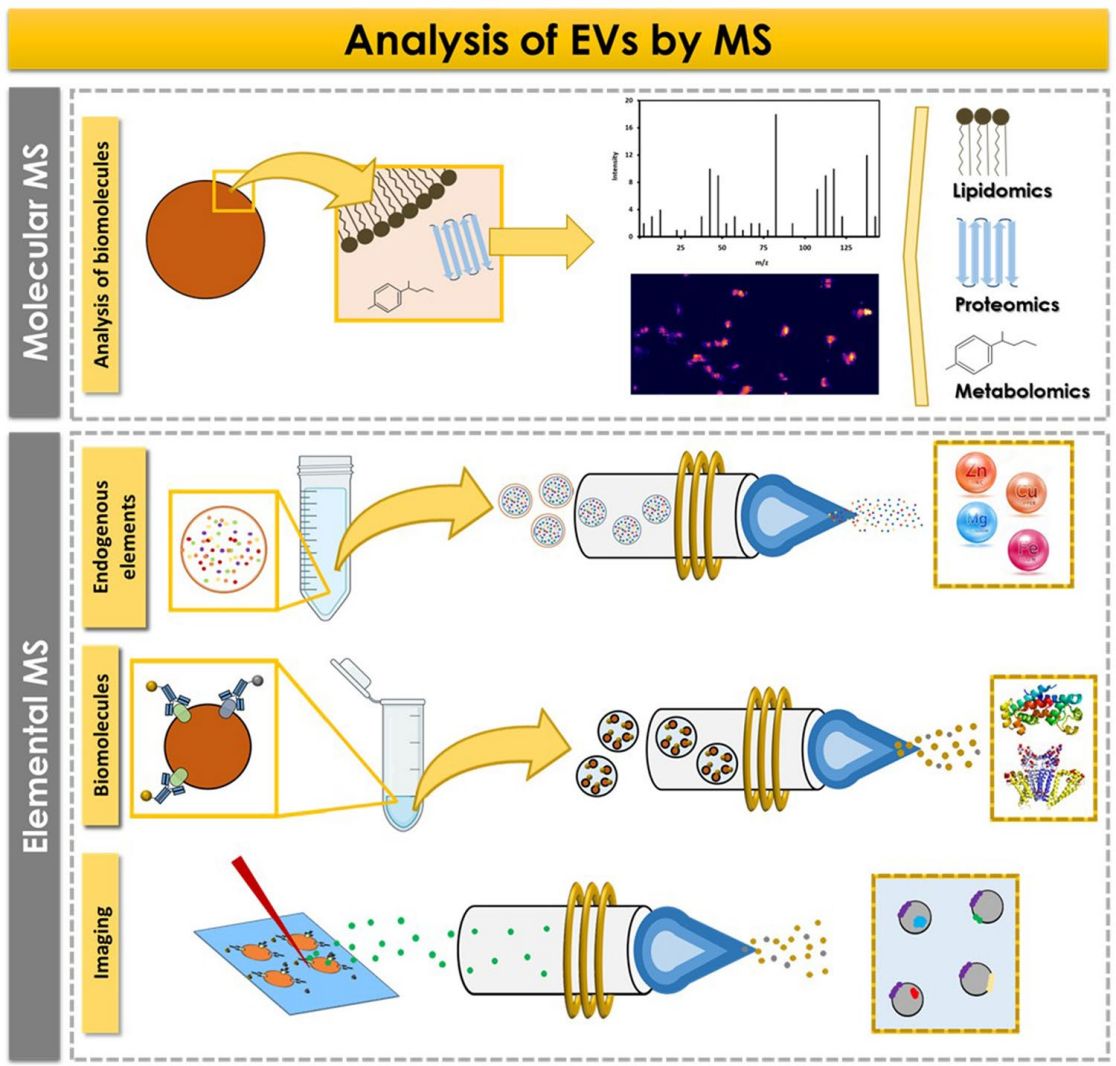
Schematic diagram illustrating the two strategies based on MS currently employed for the analysis of EVs. Molecular MS can be employed for lipidomic, proteomic, and metabolomic studies, providing valuable insights into the composition of EVs at the molecular level. On the other hand, elemental MS approaches offer information on endogenous elements, biomolecules, and distribution of target analytes within EVs

MS-based techniques offer valuable insights into EVs heterogeneity and contribute to a deeper understanding of their roles in various biological processes. We can distinguish two main MS strategies (see Fig. 2): molecular MS for performing a cargo screening and quantification of EVs biocomponents, and elemental MS for determination of endogenous elements and biomolecules (in the latter case, a previous labelling step with an elemental tag is required).

Concerning molecular MS, in the biomedical field, multi-omics approaches are being widely implemented, including proteomics, lipidomics, and metabolomics, requiring cutting-edge molecular MS-based analytical techniques, following untargeted and targeted strategies. The gold standard technique for untargeted molecular analysis of non-volatile compounds is liquid chromatography coupled to high-resolution MS. Conventional “omics” studies focus on untargeted proteomics, allowing the identification of a huge number of proteins in the sample [20]. Additionally, lipidomics and metabolomics approaches have been also reported, providing information about specific metabolic pathways in EVs genesis and in cells through their secretory activity. For example, lipidomics allows for assessment of sample purity [14] and monitoring of specific metabolic pathways involving lipids [21], whereas metabolomics contributes to understanding the role of secretory activity in cell dyshomeostasis processes associated with diseases [22]. The use of other configurations of MS instruments is also reported: matrix-assisted laser desorption/ionization (MALDI) coupled to MS is employed to identify novel biomarkers for early diagnosis, follow-up, or drug resistance prediction [23]. Imaging MALDI-MS has been also used for profiling protein biomarkers on the surface of exosomes using AuNPs-based signal amplification: exosomes from different cells subtypes were differentiated by the level of surface protein biomarkers [24].

### Elemental MS

The domain of ICP-MS has seen a significant expansion due to emerging technologies and methodologies, leading to various aspects with unique applications in research, clinical translation, and the potential for novel avenues in therapy, diagnostics, and pharmaceutical development. Moreover, it is not limited to elemental analysis alone; the combination of immunochemistry techniques with ICP-MS forms a potent tandem, extending the applicability of ICP-MS to a wide array of biomolecules [25]. These approaches have served as inspiration for mass cytometry and imaging mass cytometry.

Conventional nebulization ICP-MS stands out as a highly versatile tool for trace element research, owing to its remarkable attributes, which encompass exceptional limits of detection (LODs), minimal matrix effects, and a broad linear dynamic range. Different mass analysers can be used in ICP-MS, such as the quadrupole, magnetic sector field, or time-of-flight (ToF). Most commercially available ICP-MS instruments use scanning analysers (e.g., quadrupole filters), allowing only the sequential determination of ions with specific mass-to-charge ratios. Nevertheless, the simultaneous determination of multiple elements is necessary in many applications and the use of ToF technology offers unique advantages for such purpose. Although last-generation ICP-ToFMS systems have proved to be versatile instruments and they have been successfully employed in different application fields, main limitations are related to the high cost of the instrumentation as well as its lower sensitivity compared to quadrupole systems, particularly when dealing with low atomic mass elements. Consequently, while the analysis of several target analytes can be simultaneously conducted by ICP-ToFMS, there may be potential limitations when analysing endogenous elements within EVs.

Over the past decade, continuous advancements in ICP-MS instruments have led to the development of single-particle (sp) ICP-MS and single-cell (sc) ICP-MS techniques [26, 27]. Both techniques address the inherent heterogeneities in particle-containing samples, whether they are NPs or cells, and operate on the same fundamental principle: each sample is manipulated in such a way that the NPs (or cells) are introduced into the ICP-MS one by one (i.e., each event provides information on the elemental composition of individual NPs or cells). In this vein, specialized sample introduction systems must be employed to maximize transport efficiency (keeping also sample integrity) and the ICP-MS acquisition time should be minimized to record these events separately. By using the simultaneous analysis capabilities of ToF analysers, it becomes possible to obtain information about different analytes for each event. sp-ICP-MS provides valuable insights into the elemental composition, concentration, and size distribution of NPs [26]. On the other hand, single-cell (sc) ICP-MS shares a similar theory and instrumentation with sp-ICP-MS, enabling the study of specific analytes in individual cells. This capability enables the quantitative assessment of the high biological heterogeneity present within a cell population [27].

Elemental MS has the potential to provide direct information about trace elements present in EVs cargo. However, during our literature search, we discovered a scarcity of reports regarding the use of ICP-MS for EVs analysis. This lack of methodologies can be attributed to the challenges associated with the limited sample volume available and the extremely low levels of analytes in EVs. To overcome the microsampling of biological fluids, different strategies have been previously proposed using ICP-MS [28]. In the case of EVs, the use of sample introduction systems with high transport efficiency, that preserve sample integrity while using low sample uptake rates (in the low μL min^−1^), and the development of isolation methods that minimize cross-EVs contamination have been proposed for the analysis of endogenous elements in EVs of CCS by conventional nebulization ICP-MS [17]. These novel approaches hold potential for a deeper understanding of the role of trace elements in cellular secretory activity and the impact of different treatments on metal homeostasis.

ICP-MS can be also a suitable technique for the indirect analysis of biomolecules in EVs. In this case, the use of biomolecules that specifically recognize the target analytes and have been previously labelled with an elemental tag is required. Metal chelates are frequently employed as labels, either as individual chelates, representing a single metal atom (e.g., DOTA), or as long polymeric chelates accounting for multiple metal atoms, providing enhanced signal amplification (e.g., MAXPAR®). The use of metallic NPs of sizes up to 60 nm is also widespread. However, it is crucial to emphasize the potential benefits of employing metal nanoclusters (NCs) with diameters ranging from 1 to 3 nm and consisting of a few hundred metal atoms per NC. As NCs present a lower size compared to NPs, they exhibit less probability for sterically blocking the specific interaction between the recognition probe (e.g., the antibody) and the target biomolecule. Thus, NCs usually present better performance for multiplexing analysis compared to larger NPs. To the best of our knowledge, only three research articles have discussed the analysis of biomolecules in EVs using ICP-MS to date. The literature reports the use of nucleic acids such as DNA, aptamers, and antibodies as recognition probes [18, 19, 29]. In all cases, high amplification strategies were proposed to achieve the quantification of EVs concentration with low LOD. The use of Fe_3_O_4_@MnO_2_ nanoflowers, nanosatellite assemblies (AuNPs as core with different lanthanides elements doped up-conversion NPs), or AuNPs-labelled DNA was proposed for the sensitive detection of exosomes. Cheng et al. [29] reported LOD as low as 1.5 particles per microliter, taking advantage of the excellent recognition ability of complementary strands and the amplification power provided by rolling circle amplification.

It should be highlighted that the most widely used strategy for indirect molecular analysis by ICP-MS using metal-labelled probes is mass cytometry, where multiplexing analysis can be performed using different labels. A commercially available instrument, CyTOF®, permits the simultaneous detection of more than 20 target biomolecules using lanthanide tags, overcoming the typical limitations of flow cytometry related to spectral interferences. However, it is important to note that while the CyTOF® can operate within a mass range from 75 to 209 m/z, the optimal sensitivity is achieved within a specific and narrower mass range (between 153 and 176 m/z, which corresponds to the mass range of lanthanides commonly used as elemental labels in various methodologies). This limitation poses certain challenges when analysing endogenous elements in extracellular vesicles. Although mass cytometry has been extensively applied for biomarkers detection in cells over the past decade, few examples can be found related to EVs [30, 31]. Finally, it is worth to note that the acquisition of high-resolution images of different EVs biomolecules may be possible using laser ablation (LA) coupled to ICP-MS, if an appropriate labelling strategy is used to ensure signal amplification. Currently, the analysis of endogenous elements in EVs by LA-ICP-MS appears to be not possible due to their low concentration levels.

## Outlook

In the previous *sections*, several examples highlighting the importance of MS-based techniques in the analysis of EVs are presented, ranging from conventional to modern approaches. However, it has become evident that there is a lack of methodologies, particularly using elemental MS, for the analysis of metals and target molecules in EVs, despite the numerous potential advantages offered by ICP-MS. This observation should serve as an encouragement for researchers, as this topic deserves urgent further research, giving the exponentially growing importance of studies related to EVs in physiopathology of disease.

It is worth noting that the applications discussed are primarily focused on the analysis of EVs at a bulk level. To the best of our knowledge, there are no protocols available specifically addressing the inherent heterogeneity nature of EVs using MS. Each cell exhibits variations in terms of expressed RNA and proteins, and secretory activity. Consequently, a disease-related process would affect each cell differently, resulting in a heterogeneous population of EVs. It is important to highlight that, when working with EVs, this heterogeneity is further magnified as each cell in the organism releases different populations of EVs [2]. Therefore, two approaches must be considered to study such heterogeneities: analysis at the single-cell and the single-vesicle level. As a crucial part of the information is lost when the natural heterogeneity is not considered, there is an urgent need to develop appropriate methodologies to probe individual EVs.

**Fig. 3:**
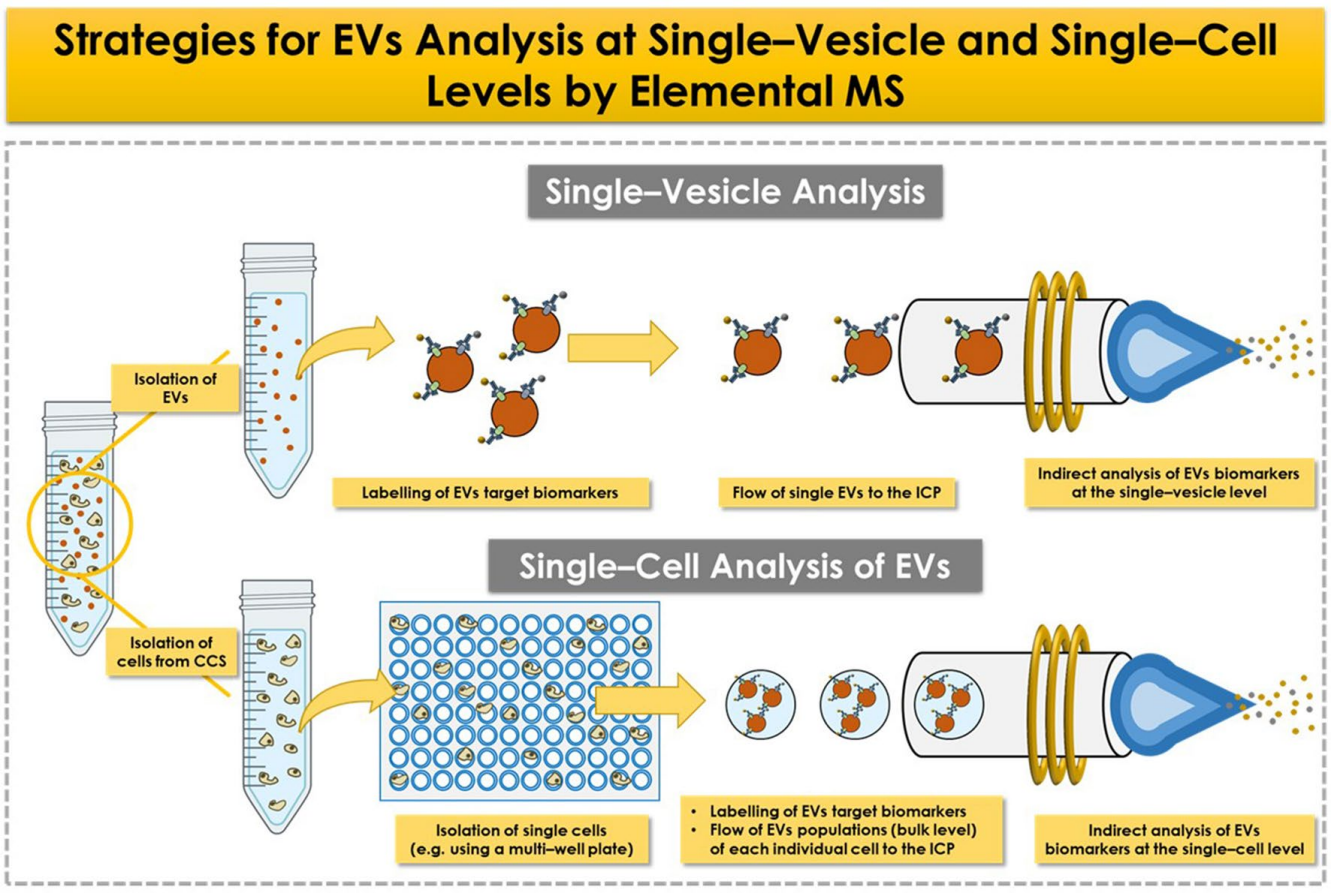
Diagram showing the two proposed approaches to address the analysis of EVs at the single-vesicle and single-cell levels using ICP-MS

Figure 3 depicts the proposed workflow for the detection of target biomolecules at the single-vesicle or single-cell level by elemental MS. In all cases, the use of high amplification labels is required to achieve enough sensitivity. For single-vesicle analysis, the methodologies already developed for sc-ICP-MS can potentially be applied, taking advantage of the structural similarities between cells and EVs [27]. However, certain challenges have to be faced, particularly related to the different dimensions of EVs and cells (nanometers and micrometers, respectively), as well as the low amount of each protein marker per vesicle. Employing small elemental labels (e.g., metal NCs) would be more favourable when compared to the currently utilized tags (such as NPs or polymeric chelates). This choice helps mitigate the potential steric hindrance that could complicate the simultaneous targeting of multiple surface antigens. Although several protocols for single-vesicle are described in the literature using flow cytometry, NTA, or digital ELISA [32], MS detection has not yet been proposed for single-vesicle analysis.

The analysis of EVs at the single-vesicle level would account for the heterogeneity within EVs populations, but not that associated with the population of cells. This can be accounted by analysing EVs at the single-cell level. In this vein, the isolation of individual cells from a cell population must be performed prior to EVs analysis, and microfluidic systems could be employed for this purpose [13]. Once cells are individually isolated and secreting EVs, an innovative strategy needs to be developed to isolate these EVs from the cells and subsequently analyse the EVs. To finish, it can be concluded that despite the numerous challenges to face for the determination of target biomolecules in EVs (bulk, as well as at the single-vesicle and single-cell level), the exceptional capabilities offered by MS-based methods pave the way for further research in this field in the coming years.
